# Computed Tomography and Magnetic Resonance Imaging of the Coronary Sinus: Anatomic Variants and Congenital Anomalies

**DOI:** 10.1007/s13244-014-0330-8

**Published:** 2014-07-22

**Authors:** Yingming Amy Chen, Elsie T. Nguyen, Carole Dennie, Rachel M. Wald, Andrew M. Crean, Shi-Joon Yoo, Laura Jimenez-Juan

**Affiliations:** 1 Department of Medical Imaging, Peter Munk Cardiac Centre, University Health Network, University of Toronto, Toronto, Canada; 2 Department of Medical Imaging, The Ottawa Hospital, University of Ottawa, Ottawa, Canada; 3 Division of Cardiology and Department of Medical Imaging, Peter Munk Cardiac Centre, University Health Network, University of Toronto, Toronto, Canada; 4 Department of Diagnostic Imaging, Hospital for Sick Children, University of Toronto, Toronto, Canada; 5 Department of Medical Imaging, Sunnybrook Health Sciences Centre, 2075 Bayview Avenue, Toronto, Ontario Canada M4N 3M5

**Keywords:** Coronary sinus anomalies, Unroofed coronary sinus, Coronary sinus ostial atresia, Coronary sinus enlargement, Coronary sinus diverticulum, Cardiac CT, Cardiac MRI

## Abstract

The coronary sinus (CS) is an important vascular structure that allows for access into the coronary veins in multiple interventional cardiology procedures, including catheter ablation of arrhythmias, pacemaker implantation and retrograde cardioplegia. The success of these procedures is facilitated by the knowledge of the CS anatomy, in particular the recognition of its variants and anomalies. This pictorial essay reviews the spectrum of CS anomalies, with particular attention to the distinction between clinically benign variants and life-threatening defects. Emphasis will be placed on the important role of cardiac CT and cardiovascular magnetic resonance in providing detailed anatomic and functional information of the CS and its relationship to surrounding cardiac structures.

*Teaching Points*

• *Cardiac CT and cardiovascular magnetic resonance offer 3D high-resolution mapping of the coronary sinus in pre-surgical planning*.

• *Congenital coronary sinus enlargement occurs in the presence or absence of a left-to-right shunt*.

• *Lack of recognition of coronary sinus anomalies can lead to adverse outcomes in cardiac procedures*.

• *In coronary sinus ostial atresia, coronary venous drainage to the atria occurs via Thebesian or septal veins*.

• *Coronary sinus diverticulum is a congenital outpouching of the coronary sinus and may predispose to cardiac arrhythmias*.

## Introduction

The coronary sinus (CS) has become an anatomic structure of increasing interest in recent years because of its importance in clinical and interventional cardiology. In diagnostic electrophysiology, the CS is commonly cannulated to record myocardial activity from the left atrium and sometimes from the left ventricle. The CS approach is also used in interventional electrophysiology for cardiac resynchronisation therapy with biventricular pacing, left-sided catheter ablation of arrhythmias and the administration of retrograde cardioplegia during cardiac surgery. However, 5-10 % of the invasive cardiac procedures fail because of unsuccessful cannulation of the CS. Causes of this include the obstruction by the valve of Thebesius as well as the presence of anatomic and congenital anatomic variants [1]. Therefore, a precise definition of the CS and recognition of the spectrum of abnormalities are crucial to ensure the safe and successful outcome of these procedures.

Cardiac CT (CCT) and cardiovascular magnetic resonance (CMR) have emerged as powerful imaging modalities in delineating the anatomy of the CS. CCT and CMR are capable of evaluating the precise morphology of the CS, including its size and relationship to adjacent structures. Both modalities contribute valuable diagnostic and management information to interventional cardiologists and surgeons accessing the CS. The strengths and weaknesses of each imaging modality are summarised in Table 1.

**Table 1:** Comparison of non-invasive imaging techniques of the coronary sinus

	Echocardiography	Cardiac CT	Cardiac MR
Spatial resolution	+	+++	+++
Temporal resolution	+++	+	++
Coronary vessel anatomy	–	+++	++
Extracardiac anatomy	+	+++	++
Ventricular function	++	+	+++
Shunt physiology	++	-	+++
Accessibility	+++	++	+
Radiation	-	+++	-
Cost	+	++	+++

## Embryology and Anatomy of the Coronary Sinus

**Fig. 1:**
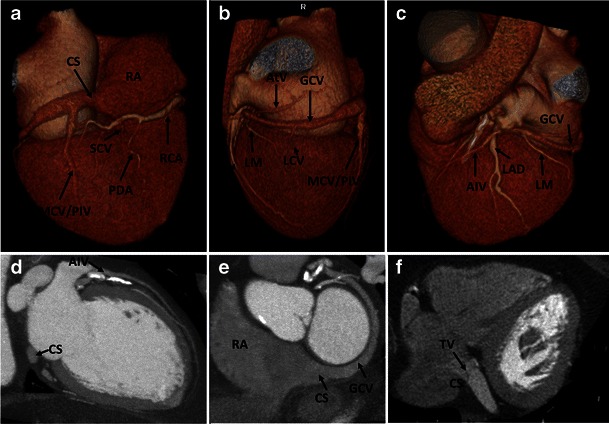
(**a**, **b**, **c**) The 3D volume-rendered and (**d**, **e**) multiplanar reformat CT images of the heart showing coronary venous anatomy. The CS is located in the posterior atrioventricular groove and opens into the right atrium via the CS ostium. It is a continuation of the great cardiac vein and collects blood from the tributary veins of both ventricles and atria. The main ventricular venous tributaries include the anterior interventricular vein, lateral cardiac vein, posterior ventricular vein (not shown), middle cardiac vein (also known as the posterior interventricular vein) and small cardiac vein. (**f**) Axial CCT images demonstrating the valve of Thebesius. RA, right atrium; CS, coronary sinus; GCV, great cardiac vein; SCV, small cardiac vein; MCV/PIV, middle cardiac vein/posterior interventricular vein; LCV, lateral cardiac vein; AIV, anterior interventricular vein; LAD, left anterior descending artery; LM, left marginal artery; RCA, right coronary artery; PDA, posterior descending artery; AtV, atrial vein; TV, valve of Thebesius

The CS is a venous conduit between the coronary veins and the right atrium, with tributary veins draining both ventricles and atria (Fig. 1). It sits posterior to the coronary sulcus, with its orifice located medial and anterior to the orifice of the inferior vena cava (IVC) and immediately above the atrioventricular junction. The orifice of a normally formed CS is always in the morphologic right atrium and is guarded by a semicircular valve, named the valve of Thebesius [2].

**Fig. 2:**
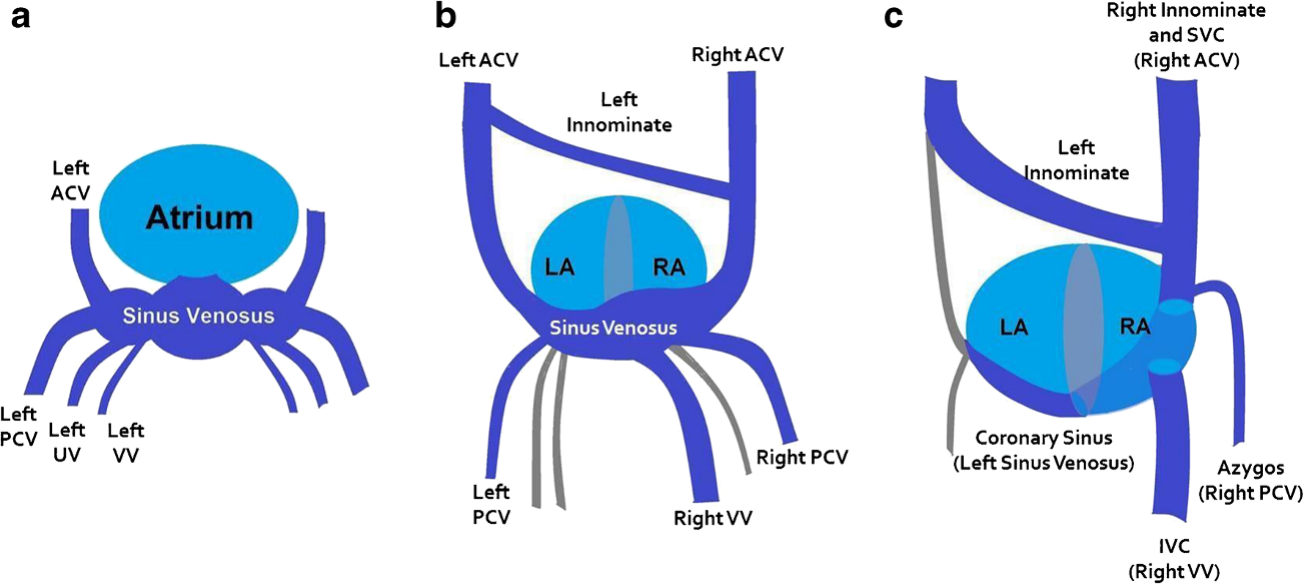
Development of the CS at 4 weeks (**a**), 7 weeks (**b**) and 8 weeks (**c**) of gestation. The sinus venosus receives three paired sets of veins. At 7 weeks, there is atrophy of the umbilical veins and the left vitelline vein (grey vessels). An oblique bridging channel forms between the left and right anterior cardinal veins, which shifts systemic venous return to the right SVC and into the right atrium. At 8 weeks, there is atrophy of the left anterior cardinal vein. The left horn of the sinus venosus develops into the CS, while the right horn becomes incorporated into the wall of the right atrium (as the sinus venarum). ACV, anterior cardinal vein; PCV, posterior cardinal vein; UV, umbilical vein; VV, vitelline vein; SVC, superior vena cava; IVC, inferior vena cava; LA, left atrium; RA, right atrium

The structural and functional significance of CS anomalies is best understood with consideration of its embryological origins. During the 8th week of development, the left innominate vein forms an oblique bridging connection between the right and left anterior cardinal veins, resulting in the shift of systemic venous return to the right superior vena cava (SVC) and into the right atrium. The left anterior and common cardinal veins involute to form the vein of Marshall, also named the oblique vein of the left atrium. The left horn of the sinus venosus becomes incorporated into the posterior right atrial wall to become the CS [2] (Fig. 2).

The CS is between 30 to 50 mm long and 10 mm wide in 75 % of adults, but there is great variation in its anatomy, including position, form, length and diameter [3].

## Imaging Modalities

Conventional coronary angiography has traditionally been used to delineate the cardiac venous anatomy. Its main limitations include the lack of three-dimensional (3D) data reconstruction and visualisation of the surrounding cardiac structures, both of which are overcome with cross-sectional imaging techniques, including CCT and CMR.

### Echocardiography

Echocardiography remains the first-line imaging modality to study the heart. However, direct visualisation and characterisation of CS and associated anomalies are often difficult and unsatisfying on transthoracic echography, as CS and related venous vasculature are posterior structures without an intervening acoustic window to allow sound penetration.

The most obvious clue on echocardiography alerting cardiologists to the presence of a CS anomaly is the presence of a dilated CS. Agitated bubble saline contrast injected through the left antecubital vein can indicate the presence of a persistent left SVC (LSVC) draining to the CS [4]. Visualisation of bubble contrast in the left atrium before the right atrium is suggestive of the presence of a right-to-left shunt.

### Cardiac CT

The CCT technique for CS imaging is similar to that for routine coronary CT angiography, with some modifications. Table 2 provides a description of the CCT parameters used in our institution. If the heart rate is stable and below 65 bpm, a prospective ECG-gated CT is preferred to reduce the radiation dose. To optimise the visualisation of the CS, both bolus test injection and bolus tracking can be used. Our institution uses bolus test injection, with image acquisition timed to maximal CS opacification, which usually occurs 4 s after coronary arterial opacification. Using bolus tracking, the automated trigger level should be placed in the descending aorta, and image acquisition should start 4 s after reaching a threshold of 180 Hounsfield units [5]. In patients who present with a poor ejection fraction (a frequent scenario in those considered for resynchronisation therapy), CS opacification can be optimised by delaying the scan start time. At our institution, the contrast volume and injection rate are adapted to body weight. All available techniques are applied to keep the radiation dose as low as possible. Contrast injection through the left arm is suggested when attempting to show anomalous LSVC connections.

**Table 2:** Cardiac CT parameters in use at our institution

Contrast volume/injection rate^a^	<60 kg: 60 cc @ 4 cc/s 60–80 kg: 85 cc @ 5 cc/s 80–100 kg: 95 cc @ 6 cc/s >100 kg: 105 cc @ 7 cc/s
Voltage (kV)	<60 kg: 100–120 kV 60–100 kg: 120 kV >100 kg: 135 kV
Current (mA)	BSA ≤ 18.5: 120 kV@370 mA BSA 18.5-25: 120 kV@ 440 mA BSA 25–30: 120 kV@510 mA BSA >30: 120 kV@580 mA or 135 kV @510 mA
Test bolus	To test timing 4 s after maximum opacification of the coronary arteries
Coverage	160 mm Volume (0.5 × 320 mm)
Tube rotation	0.35 s
Scan mode	Prospective gating for: HR < 60 bpm: 70-80 % HR 60–63 bpm: 60 % - R HR 63–65 bpm: 40 % - R Retrospective triggering for: HR > 65

^a^All injections are followed by a 20-cc saline chaser at the same injection rate

### Cardiovascular Magnetic Resonance

CMR has emerged as a powerful and versatile modality in detecting structural and functional abnormalities of the heart, providing accurate quantification of ventricular size and function, as well as shunt volume using velocity-encoded cine sequences. The more recent development of specialised sequences described below has extended the use of CMR in delineating the coronary venous system [6]. These sequences offer high anatomical detail of small vascular branches with no ionising radiation, particularly suitable for imaging of children and young adults [7].

The proposed imaging protocol includes steady-state free procession (SSFP) cine imaging for the assessment of the cardiac anatomy, acquired in different standardised planes, including axial, 2-, 3- and 4-chamber and short axis oblique planes covering the atria. This is followed by dynamic time-resolved contrast-enhanced 3D MR angiography in venous phase to delineate the often complex course of extracardiac vascular branches. More detailed assessment of the coronary venous vasculature can be obtained using a 3D SSFP sequence with or without intravenous contrast agents for the simultaneous evaluation of both coronary arteries and veins, especially as part of pre-interventional procedural planning [8]. Since the coronary vein diameter is larger in systole, a systolic acquisition has been proposed [6]. Finally, phase-contrast imaging provides quantitative evaluation of shunt physiology and relates anatomic morphology to functional significance [9].

## Anomalies of the CS

### Acquired

**Fig. 3:**
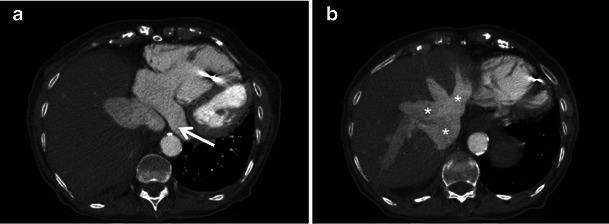
A 75-year-old male with severe tricuspid regurgitation. (**a**, **b**) Axial chest CT images demonstrate severe right atrial and ventricular enlargement secondary to severe tricuspid regurgitation. Right heart volume and pressure overload led to severe dilatation of the CS (*arrow*), IVC and hepatic veins (*asterisks*)

The most common type of acquired CS anomaly is CS enlargement, which results from conditions with raised right heart pressure, including valvular dysfunction, chronic volume overload and pulmonary arterial hypertension (Fig. 3).

### Congenital

Congenital enlargement of CS is caused by increased blood flow into the CS via anomalous connections and can be divided into two broad categories based on the presence or absence of a left-to-right shunt [10].

#### Enlargement of CS without Left-to-Right Shunt

**Fig. 4:**
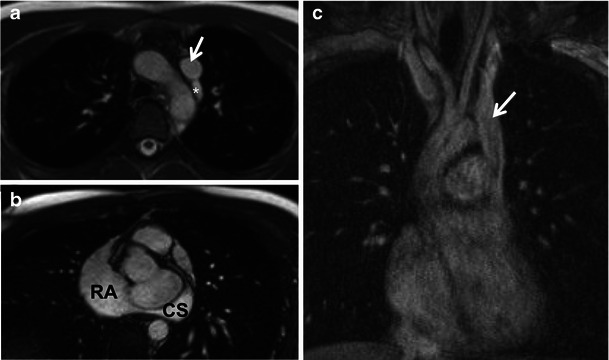
A 17-year-old male with absent right SVC and persistent LSVC draining into the CS. **a** Axial and **b** short-axis cine balanced SSFP images and **c** coronal maximum intensity projection image from thoracic MR angiography demonstrating a persistent LSVC (**a**, **c**, *arrow*) draining into the right atrium (**b**, RA) via the CS (**b**). The azygous vein is left sided and drains into the persistent LSVC (**a**, asterisk)

CS enlargement without shunt is a physiologically benign entity and occurs secondary to the presence of a persistent LSVC or, more rarely, partial anomalous hepatic venous connection to the CS [10]. Embryologically, a persistent LSVC arises because of failure of regression of the left anterior cardinal vein into the ligament of Marshall. The LSVC receives blood from the left upper extremities and head and neck structures, descends anterior and lateral to the aortic arch, courses obliquely along the posterior wall of the left atrium and joins the CS in the posterior AV groove before draining into the right atrium [3]. In 10 % of cases, persistent LSVC is associated with the agenesis of the right SVC, which may further dilate the CS because of increased venous return (Fig. 4) and may result in CS aneurysm formation [11, 12].

The diagnosis of CS enlargement associated with persistent LSVC has largely been made incidentally on imaging or during surgery for associated cardiac defects. Although an enlarged CS with persistent LSVC is benign in isolation, it is often associated with other congenital heart defects, including an atrial septal defect (ASD), ventricular septal defect (VSD), atrioventricular septal defect (AVSD), pulmonary stenosis and cor triatriatum [10, 13]. Furthermore, persistent LSVC with CS enlargement may affect the development of cardiac conduction tissue, with reported association with atrial arrhythmias [14].

#### Enlargement of CS with Left-to-Right Shunt: Unroofed CS

**Fig. 5:**
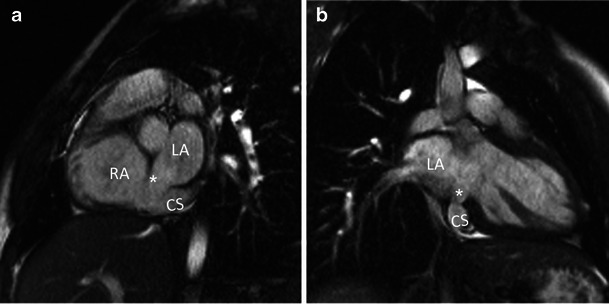
Unroofed CS in a 9-year-old male. Cine balanced SSFP images in short-axis **a** and two-chamber planes **b** show a defect (*asterisk*) in the wall between the CS and the left atrium near the CS ostium

**Fig. 6:**
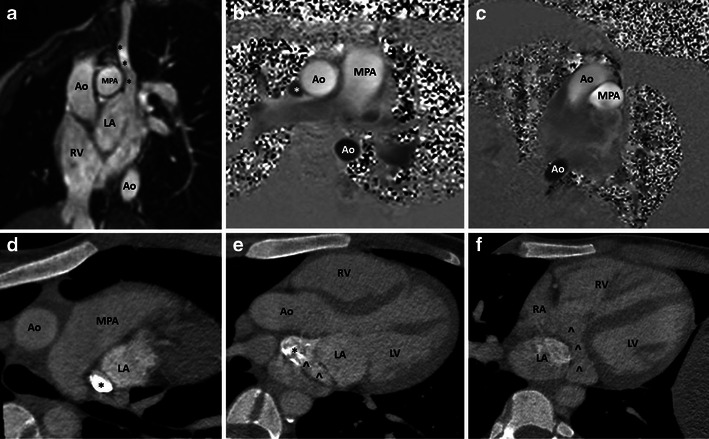
A 21-year-old female with partial unroofing of the coronary sinus and persistent LSVC. Oblique cine balanced SSFP image (**a**) shows the communication of the LSVC and the left atrium. Phase-contrast mapping of the main pulmonary artery (**b**) and aorta (**c**) indicates net forward flow of 130 ml and 80 ml per beat respectively, giving rise to a Qp:Qs ratio of 1.6:1. The phase-contrast image also demonstrates caudal blood flow in the LSVC (*). Serial reconstructed CCT images (**d**, **e**, **f**) show contrast leak from the LSVC (*) into the left atrium before reaching the right atrium via the CS (^), indicating partial unroofing. *, left superior vena cava; ^, coronary sinus; LA, left atrium; LV, left ventricle; RA, right atrium; RV, right ventricle; Ao, aorta; MPA, main pulmonary artery

**Fig. 7:**
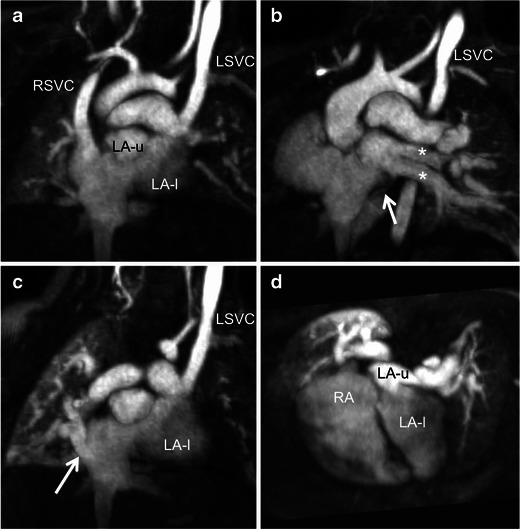
Completely unroofed CS with persistent LSVC and cor triatriatum in a 13-month-old male. Multiplanar maximum intensity projection MR angiography images show bilateral SVC with no bridging vein, cor triatriatum and scimitar vein. Two left pulmonary veins (**b**, asterisks) and a tiny vein draining the medial aspect of the right lower lung (**b**, arrow) connect to the upper chamber of the divided left atrium (LA-u). A scimitar vein draining most of the right lung connects to the junction between the right atrium and IVC (**c**, arrow). The persistent LSVC connects to the roof of the lower chamber of the left atrium (LA-l). There is complete unroofing of the CS into the left atrium. The CS ostium behaves as a large interatrial communication between the lower chamber of the left atrium and the right atrium, i.e. a CS ASD. Surgical management involved removing the membrane dividing the left atrium and creating a tunnel between the persistent LSVC and the right atrium using an autologous pericardial patch

CS enlargement with left-to-right shunt is most commonly caused by an anomalous communication between the CS and the left atrium, in the form of a partially or completely “unroofed” CS (Figs. 5 and 6). An unroofed CS can occur in isolation or in association with a persistent LSVC. The functional significance of an *isolated* unroofed CS defect is the same as an ASD. However, shunt physiology in an unroofed CS defect *with LSVC* can be more complex. In the most common scenario, there is a small right-to-left shunt at the LSVC-left atrial connection and a relatively greater left-to-right shunt at the unroofed CS-left atrial connection, resulting in a net left-to-right shunt. However, the net direction of the shunt reverses if the right atrial pressure becomes abnormally elevated, for example in the case of pulmonary arterial hypertension [15] or atrioventricular valve atresia/stenosis [16]. Right-to-left shunts secondary to persistent LSVC and unroofed CS have been reported to cause cerebral abscesses [17]. Multiple congenital heart defects have also been reported in association with unroofed CS, including atrioventricular valve atresia, ASD, VSD, anomalous pulmonary venous connection, cor triatriatum (Fig. 7) and tetralogy of Fallot [18, 19].

**Fig. 8:**
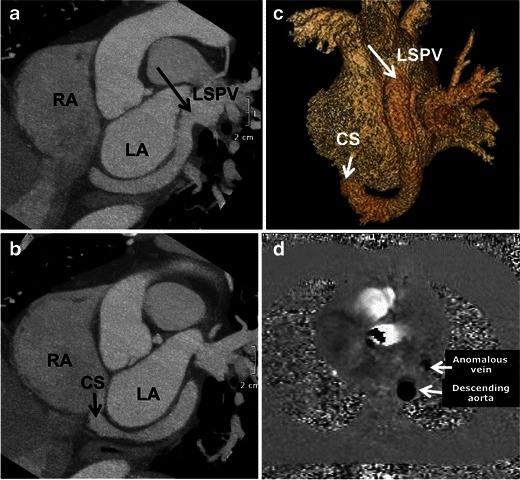
A 53-year-old male with an anomalous communication of the left superior pulmonary vein (LSPV) to the CS. (**a**, **b**) Short axis maximum intensity projection CCT images and (**c**) volume-rendered image clearly delineate the course of the CS in the atrioventricular groove posterior to the left atrium, receiving drainage from the LSPV and opening into the right atrium. (**d**) CMR with phase-contrast analysis demonstrates the same caudal direction of flow of the anomalous vein and the descending aorta

More rarely, anomalous communication occurs between the CS and the pulmonary veins (Fig. 8). In anomalous pulmonary connection to the CS, some or all of the pulmonary veins join a confluence that connects with the CS, resulting in oxygenated and deoxygenated blood mixing in the right atrium [20, 21]. An ASD is required to survive in the case of total anomalous pulmonary venous return.

#### Enlargement of CS with High Pressure Left-to-Right Shunt: Fistulous Connection between CS and Coronary Arteries

**Fig. 9:**
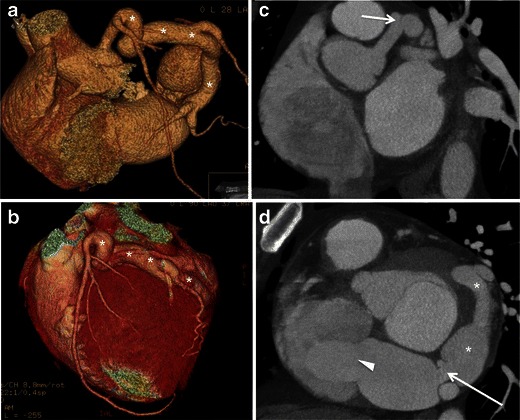
A 50-year-old male with fistulous connection between the left circumflex artery and the CS. (**a**, **b**) Volume-rendered reformats and (**c**, **d**) short axis maximum intensity projection CCT images demonstrate enlargement of the left circumflex (*asterisks*) from its origin (**c**, *arrow*) to its connection to the CS via a small neck (**d**, *arrow*). The CS is markedly enlarged and arterialised (**d**, *arrowhead*)

Coronary artery fistula to the CS leads to marked dilation of both vessels (Fig. 9) and embryologically results from persistent intratrabecular sinusoids between the coronary arteries and the CS [22].

Patients with coronary artery fistula to the CS are at increased risk of myocardial infarction, progressive heart failure and infective endocarditis [22], and may experience aneurysm-related complications including left atrial compression (i.e. pseudo-mitral valve stenosis), thrombus formation, and aneurysm dissection and/or rupture [22, 23]. In these patients undergoing cardiac surgery, myocardial protection is poorly achieved because of run-off of cardioplegic solution into the right atrium [24].

Surgical repair is indicated if the patient is symptomatic or presents with aneurysm-associated complications, which involves ligation or embolisation, with surgical enlargement of the CS ostium if necessary [22]. Surgical planning relies increasingly on the excellent spatial mapping capacity of coronary CT angiography and MR angiography.

#### CS Ostial Atresia/Stenosis

**Fig. 10:**
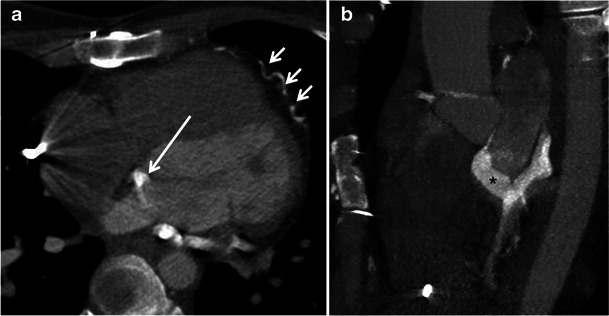
A 26-year-old female with Ebstein anomaly, CS ostial atresia and a persistent LSVC draining into the left atrium via the CS. **a**, **b** Multiplanar reformat CT images demonstrate communication between the CS and the left atrium (**a**, *long arrow*) via an anomalous vein (**b**, *asterisk*) without connection to the right atrium. Coronary venous drainage occurs through a network of collateral veins feeding directly into the right atrium (**a**, *short arrows*)

**Fig. 11:**
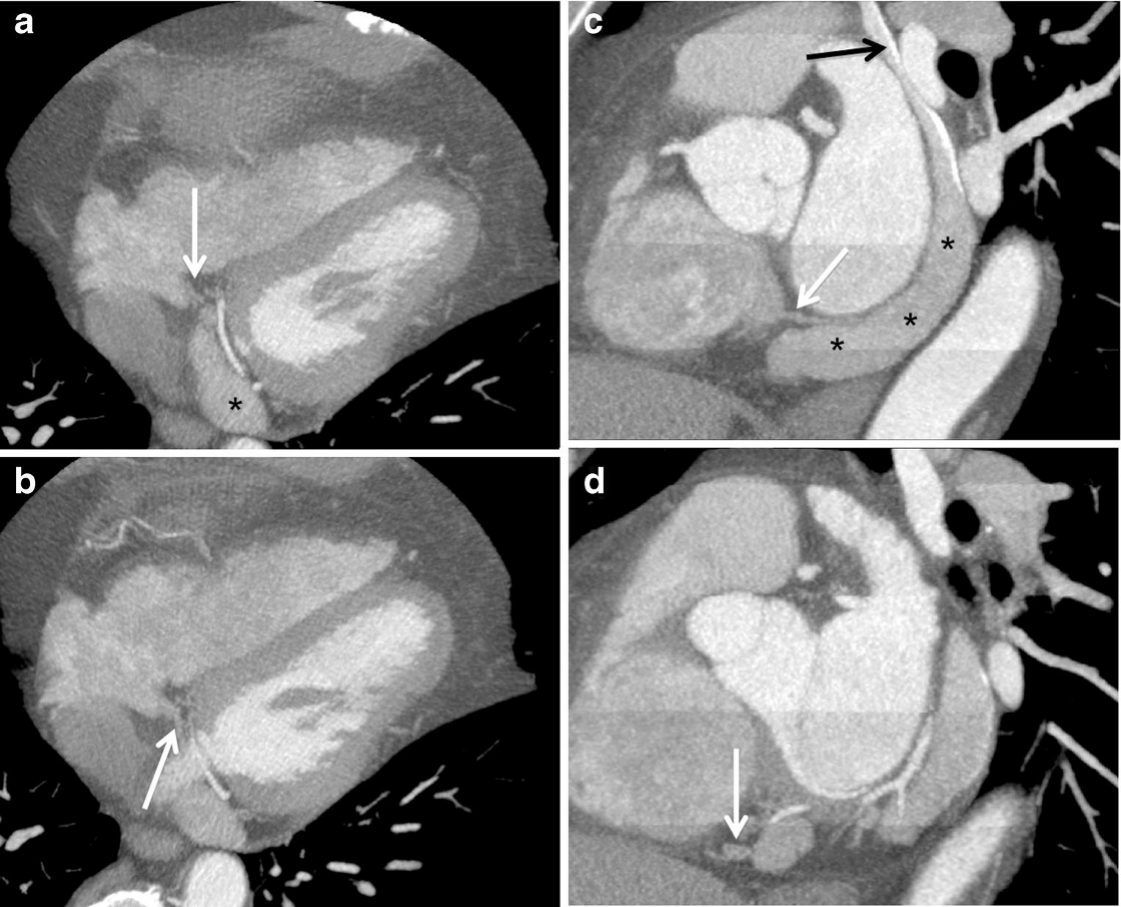
A 32-year-old male with CS ostial atresia and a persistent LSVC. Multiplanar maximum intensity projection CCT images demonstrate the blind-ending sac of the CS (**a**, **b**, *arrows*) and associated CS dilatation (**a**, **c**, *asterisks*). Note the small dilated Thebesian and interatrial septum veins draining into the right atrium (**c**, **d**, *white arrows*). Intravenous contrast was injected through the right arm and there is some dense contrast in the persistent LSVC (**c**, *black arrow*), which may be due to a bridging vein (not imaged)

In CS ostial atresia, the CS lies in a normal position but ends as a blind sac, thus preventing usual drainage into the right atrium. A significant proportion of individuals with CS ostial atresia have CS dilatation (diameter greater than 12 mm). Coronary venous drainage may occur via collateral communications between the CS and the right atrium, including multiple enlarged thebesian veins, interatrial septal veins or an unroofed CS into the left atrium [25] (Fig. 10). When a persistent LSVC is present, the coronary venous blood can also flow in a retrograde (cephalad) direction up the LSVC into the left innominate vein, then into the right atrium [26] (Fig. 11).

Although most cases of CS ostial atresia are physiologically asymptomatic, lack of recognition of the anomaly during cardiac surgery can lead to fatal consequences. Ligation of LSVC leads to acute venous obstruction and myocardial congestion and ischaemia [27]. Patients with LSVC and narrowed/atretic CS ostium also present difficulties in introducing pacemaker or defibrillator leads [28]. CMR has become crucial in evaluating the direction of flow in incidental LSVC and ensuring the patency of CS prior to surgical manipulation [28]. Surgical repair of CS ostial atresia involves unroofing of the CS into the right or left atrium.

#### Absence/Hypoplasia of CS

Complete absence of CS usually occurs with persistent LSVC terminating in the left atrium with an ASD situated in the posteroinferior angle of the atrial septum at the position normally occupied by the CS ostium (i.e. CS ASD) [10, 19]. Absence of CS has also been reported to occur in isolation [29]. In both scenarios, coronary veins empty individually into their corresponding atria.

#### CS Diverticulum

**Fig. 12:**
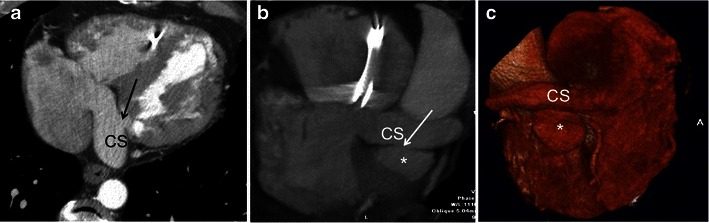
A 50-year-old male with repaired tetralogy of Fallot and CS diverticulum. He has a pacemaker. **a** Axial CT, **b** maximum intensity projection and **c** volume-rendered CCT images demonstrate a congenital diverticulum (**b**, **c**, asterisks) arising from the CS, with a narrow neck (**a**, **b**, arrow). The diverticulum lies between the CS and the mitral valve annulus. The CS is enlarged, which could be a source of arrhythmia

CS diverticulum is a congenital outpouching of the CS, typically with a distinct neck that extends behind the left ventricle. The wall of the diverticulum, particularly of its neck region, is in close proximity to the posteroseptal and left posterior accessory conduction pathways and thus predisposes the affected individual to cardiac arrhythmias and sudden cardiac death [30, 31]. The anomaly is often diagnosed incidentally on CS venography during electrophysiological studies and catheter ablation of the accessory pathway [32]. CCT (Fig. 12) and CMR with cine imaging accurately define the anatomy of the CS diverticulum and adjacent structures, while dynamic time-resolved contrast-enhanced 3D MR angiography sequences demonstrate timing of contrast flow into the diverticulum [30, 32, 33].

## Conclusion

CS anomalies are becoming increasingly recognised because of their relevance in interventional cardiology and association with other congenital cardiac defects. CS enlargement is the most common type of CS anomaly and may be clinically benign without shunt, or clinically symptomatic in the presence of shunt from an unroofed CS into the left atrium or fistulous connection between the CS and the coronary arteries. Some anomalies, such as CS ostial atresia, may be physiologically benign but may have fatal consequences if unrecognised prior to surgical manipulation. Others, such as CS diverticulum, may present with cardiac arrhythmias and sudden cardiac death. CMR and CCT have become standard imaging modalities in guiding the diagnosis and treatment of CS anomalies because they provide high-resolution anatomic information about the morphology and course of the CS. CMR has the advantage of avoiding ionising radiation but with lower spatial resolution compared to CCT. Information obtained from these non-invasive imaging modalities can help identify and avoid potential complications during cardiac interventions.
